# Occurrence of occupational disease among basic education teachers in Minas Gerais, Brazil

**DOI:** 10.47626/1679-4435-2025-1511

**Published:** 2025-12-09

**Authors:** Daiane Ferreira Mendes, Maria Clara Silva dos Santos, Rose Elizabeth Cabral Barbosa, Amanda Mota Lacerda, Giovanni Campos Fonseca, Desirée Sant’Ana Haikal

**Affiliations:** 1 Dentistry, Universidade Estadual de Montes Claros (Unimontes), Montes Claros, MG, Brazil; 2 Graduate Program in Health Sciences, Unimontes, Montes Claros, MG, Brazil; 3 Instituto de Ciências Agrárias, Universidade Federal de Minas Gerais, Campus Montes Claros, MG, Brazil

**Keywords:** professores escolares, doenças profissionais, saúde ocupacional, inquéritos epidemiológicos., schoolteachers, occupational diseases, occupational health, health surveys.

## Abstract

**Introduction:**

Working conditions in teaching - encompassing physical, mental, and emotional demands - may overload psychophysiological functions.

**Objectives:**

To analyze the occurrence of self-reported diagnosis of occupational disease and associated factors among public basic-education teachers in Minas Gerais, Brazil.

**Methods:**

Cross-sectional epidemiological study using data from the follow-up phase of the longitudinal project titled “Health and working conditions of basic education teachers in the state of Minas Gerais” (ProfSMinas). The dependent variable was self-reported diagnosis of an occupational disease. Explanatory variables included sociodemographic characteristics, work and employment conditions, health status, and lifestyle factors. Binary logistic regression was employed to estimate crude and adjusted odds ratios with 95%CIs.

**Results:**

The prevalence of self-reported diagnosis of an occupational disease was 24.1% (95%CI 22.6%-25.6%). Positive associations were observed for age, student-perpetrated physical and/or verbal violence, job dissatisfaction, use of antidepressants and/or sedatives, sickness absence, and suspected voice disorders.

**Conclusions:**

Multiple factors contribute to occupational illness among teachers, underscoring the need for ongoing studies to inform prevention and health-promotion strategies.

## Abstract (pt)

**Introdução:**

As condições de trabalho às quais os professores são expostos - envolvendo demandas físicas, mentais e emocionais impostas pela atividade docente - podem sobrecarregar funções psicofisiológicas.

**Objetivos:**

Analisar a ocorrência de diagnóstico de doença ocupacional e seus fatores associados entre professores da educação básica da rede pública estadual de Minas Gerais, Brasil.

**Métodos:**

Este estudo epidemiológico transversal com dados da fase de seguimento do Projeto “Condições de saúde e trabalho de professores da educação básica do estado de Minas Gerais: estudo longitudinal” (ProfSMinas). A variável dependente foi o autorrelato de diagnóstico de doença ocupacional. As variáveis explicativas incluíram características sociodemográficas, de trabalho e emprego, situação de saúde e estilo de vida. Empregou-se regressão logística binária; as associações brutas e ajustadas foram estimadas por *odds ratios* e respectivos intervalos de confiança de 95% (IC95%).

**Resultados:**

A ocorrência de relatos de diagnóstico de doença ocupacional foi de 24,1% (IC95% 22,6%-25,6%). Houve associação com idade, violência física e/ou verbal praticada por estudantes, insatisfação com o trabalho, uso de antidepressivos e/ou calmantes, absenteísmo por doença e suspeita de distúrbios vocais.

**Conclusões:**

Diferentes fatores influenciam o adoecimento docente por doenças ocupacionais, indicando a necessidade de estudos contínuos para orientar ações de prevenção e promoção da saúde.

## INTRODUCTION

Work is a key determinant of individual well-being and social belonging.^1^ Conversely, under certain circumstances, it can become a central risk factor for illness.^2^ Occupational diseases are currently defined as conditions that affect workers when the work environment or working conditions significantly influence the illness process, with varying levels of intensity.^1^

Professionals who work directly with the public - such as teachers, health workers, and police officers - tend to experience the professional demands of their roles more intensely.^3^ In education, mismatches between demands and available resources, as well as between expectations and reality, are common; overload and imposed requirements often exceed the physical and cognitive capacities of these professionals.^4^

Within this context, the ways in which work directly affects teachers’ health warrant attention. Working conditions under which teachers employ their physical, mental, and emotional capacities to meet school production goals and diverse demands may lead to overload or excessive use of psychophysiological functions.^5^

Changes in the education system aimed at expanding access and updating curricula have also transformed physical and organizational aspects of the school environment,^6^ including increases in enrollments, class groups, and students per classroom, thereby intensifying teachers’ workloads.^7^ In addition, continuous progression - advancing students despite isolated failures - particularly in the early years of public elementary education, may require methodological and content adaptations for classes with heterogeneous knowledge levels, heightening pressure on teachers to ensure learning objectives are met.^8^ This scenario favors the emergence of anxiety, depression, stress, and other occupational conditions.^6^

These conditions contribute to clinical symptoms that help explain high rates of work absence.^9^ Their impacts translate into substantial social and financial costs, reflected in individual suffering, productivity losses, and increased strain on health services.^10^ Teacher absenteeism may also function as a defensive mechanism related to the occupational stress inherent to pedagogy, aimed at preserving psychological well-being.^5^ Socioeconomically, it generates substitution costs and compromises education quality; it weakens pedagogical bonds, hinders achievement of educational goals, and strains interpersonal relations, resulting in services that fall short of expectations. Ineffective management of short absences, which often preclude replacement, overburdens present staff and may intensify professional conflicts.^11^

Underreporting of occupational morbidities - such as dysphonia - limits formal recognition of these conditions, hinders access to social security benefits, and raises overall costs of the process.^9^ More broadly, occupational risks lead to substantial economic losses, estimated at 4%-6% of gross domestic product in most countries.^11^

The aim of this study was to analyze the occurrence of a diagnosis of occupational disease and associated factors among public basic-education teachers in the state of Minas Gerais.

## METHODS

We conducted an analytical, cross-sectional web-based survey during the follow-up phase of the longitudinal project “Health and working conditions of basic-education teachers in the state of Minas Gerais” (ProfSMinas), in accordance with the Checklist for Reporting Results of Internet E-Surveys.^12^

The target population comprised approximately 90,000 basic-education teachers (early childhood, elementary, and secondary education) working in about 3,400 state public schools, according to the Secretariat of Education of the State of Minas Gerais (SEE-MG) payroll for July 2021.

For the baseline, the sample size was calculated assuming 50% prevalence for the outcome, an infinite population, a 3% margin of error, and a 20% increment for anticipated losses, yielding 1,282 teachers to ensure statewide representativeness. For the follow-up, an electronic questionnaire was sent to all 1,907 baseline participants. Data collection occurred from October 2023 to March 2024. Eligible participants were those who, at the time of data collection, held a teaching position in a state public school.

The dependent variable was the self-report of a medically diagnosed occupational disease, obtained from the question: “Have you ever had an occupational or work-related disease (diagnosed by a physician)?” Responses were categorized as yes/no.

We evaluated i) sociodemographic characteristics: sex (female; male), age (≤ 39 years; 40-49 years; ≥ 50 years), and educational attainment (postgraduate degree: yes; no); ii) work and employment characteristics: years of teaching (≤ 10; 11-20; ≥ 21), employment type (tenured/civil service; temporary/contracted), weekly workload (≤ 39 hours; ≥ 40 hours), excessive classroom noise (no; yes), classroom indiscipline (no; yes), episodes of physical and/or verbal violence (no; yes), effort-reward imbalance (no; yes), overcommitment to work (no; yes), and job dissatisfaction (no; yes); iii) health status and lifestyle: self-rated health (positive; negative), self-rated quality of life (positive; negative), diagnosis of a noncommunicable chronic disease (no; yes), diagnosis of depression and/or anxiety in the previous 12 months (no; yes), psychological/psychiatric follow-up (no; yes), use of antidepressants and/or tranquilizers (no; yes), sickness absence in the previous 12 months (no; yes), poor sleep quality (no; yes), suspected voice disorders (no; yes), healthy eating pattern (no; yes), and regular physical activity (no; yes).

Effort-reward imbalance and overcommitment were assessed using the short version of the Effort-Reward Imbalance Questionnaire.^13^,^14^ The imbalance index was calculated as (e/r)×c, where *e* is the sum of effort items, *r* the sum of reward items, and *c* a correction factor for the different numbers of items; values > 1 indicate imbalance (effort exceeds reward). Overcommitment was dichotomized at the median (yes: ≥ median; no: < median). Self-rated health was obtained from the question, “How satisfied are you with your health?” Responses “satisfied/very satisfied” were grouped as positive, and “very dissatisfied/dissatisfied/neither satisfied nor dissatisfied” as negative. Quality of life was assessed by the question, “How do you rate your quality of life?” Responses “good/very good” were grouped as positive, and “very poor/poor/neither poor nor good” as negative.

Presence of noncommunicable chronic disease considered physician-diagnosed hypertension, diabetes, and/or heart or vascular disease.^15^

Sleep quality was assessed with the Pittsburgh Sleep Quality Index^16^; the total score is the sum of seven components, and values > 5 indicate poor sleep quality. Suspected voice disorder was measured with the Screening Index for Voice Disorder^17^; 1 point is assigned to each symptom marked “Always” or “Sometimes,” and scores ≥ 5 indicate suspicion of a disorder. Dietary pattern was measured with the Scale for Measuring Healthy Eating Practices,^18^ which covers meal planning, household organization, eating behaviors, and food choices aligned with the Brazilian Dietary Guidelines; scores ≥ 41 defined a healthy pattern (yes) and < 41 an unhealthy pattern (no). Regular physical activity was obtained using the International Physical Activity Questionnaire (IPAQ), considering frequency and duration of vigorous and moderate activities and walking; individuals classified as “active” or “very active” were considered regular practitioners.^19^

Analyses were performed in Stata 13.0 (StataCorp LLC, College Station, TX). We first described the sample by sociodemographic, work/employment, health status, and lifestyle characteristics. We then estimated the prevalence of a diagnosis of occupational disease in the overall sample and across categories of explanatory variables. Bivariate associations were examined using binary logistic regression, yielding crude odds ratios (ORs) with 95% CIs. Variables with p ≤ 0.20 were entered into the multivariable model. In the multiple analysis - also via binary logistic regression - we applied manual backward elimination, sequentially removing variables until only those with p ≤ 0.05 remained. We report crude and adjusted ORs with 95% CI. Model fit was assessed with the Hosmer-Lemeshow test; a higher p-value indicates better fit.

The ProfSMinas project was approved by the Research Ethics Committee at Universidade Estadual de Montes Claros (approval no. 4.964.125) and conducted with formal authorization from SEE-MG. All participants provided electronic informed consent when they accessed the questionnaire.

## RESULTS

Of the 1,907 participants at baseline, 767 teachers completed the follow-up questionnaire. The sample was predominantly female (71.3%) and had a mean age of 45.6 ± 8.9 years. Table 1 presents the detailed characteristics of the sample.

**Table 1:** Characteristics of the sample according to sociodemographic, work/employment, health, and lifestyle variables. Public basic-education teachers in Minas Gerais, 2024 (n = 767)

Variable	n	%
Sex		
Female	547	71.3
Male	220	28.7
Age (years)		
≤ 39	213	27.8
40-49	288	37.5
≥ 50	266	34.7
Postgraduate degree		
No	267	34.8
Yes	500	65.2
Years of teaching experience		
≤ 10	132	17.2
11-20	340	44.3
≥ 21	295	38.5
Employment type		
Tenured/public service	539	70.3
Temporary/contracted	228	29.7
Weekly workload (hours)		
≤ 39	468	61.0
≥ 40	299	39.0
Excessive classroom noise		
No	374	48.8
Yes	393	51.2
Classroom indiscipline		
No	361	47.1
Yes	406	52.9
Physical and/or verbal violence		
No	265	34.5
Yes	502	65.5
Effort-reward imbalance		
No	197	25.7
Yes	570	74.3
Overcommitment to work		
No	356	46.4
Yes	411	53.6
Job dissatisfaction		
No	544	70.9
Yes	223	29.1
Self-rated health		
Positive	338	44.1
Negative	429	55.9
Self-rated quality of life		
Positive	446	58.2
Negative	321	41.8
Noncommunicable chronic disease		
No	509	66.4
Yes	258	33.6
Diagnosis of depression		
No	600	78.2
Yes	167	21.8
Diagnosis of anxiety		
No	404	52.7
Yes	363	47.3
Psychological/psychiatric follow-up		
No	551	71.8
Yes	216	28.2
Use of antidepressants/tranquilizers		
No	510	66.5
Yes	257	33.5
Sickness absence		
No	365	47.6
Yes	402	52.4
Poor sleep quality		
No	185	24.1
Yes	582	75.9
Suspected voice disorder		
No	546	71.2
Yes	221	28.8
Healthy eating pattern		
No	521	67.9
Yes	246	32.1
Regular physical activity		
No	327	42.6
Yes	440	57.4

The prevalence of self-reported diagnosis of occupational disease was 24.1% (95% CI 22.6%-25.6%). In the bivariate analyses, nearly all explanatory variables were associated with the outcome at the 20% significance level, except for sex, weekly workload, self-rated health, and regular physical activity (Table 2).

**Table 2:** Prevalence of occupational disease diagnosis according to sociodemographic, work/employment, health, and lifestyle variables, and bivariate analyses. Public basic-education teachers in Minas Gerais, 2024 (n = 767)

Variable	Prevalence of occupational disease (%)	Crude OR (95%CI)	p-value
Sex			
Female	25.2	1.00	
Male	21.4	0.81 (0.55-1.17)	0.258
Age (years)			
≤ 39	19.3	1.00	
40-49	28.8	1.70 (1.11-2.60)	0.015
≥ 50	22.9	1.25 (0.80-1.95)	0.328
Postgraduate degree			
No	20.2	1.00	
Yes	26.2	1.40 (0.98-2.01)	0.066
Years of teaching experience			
≤ 10	17.4	1.00	
11-20	23.5	1.46 (0.87-2.44)	0.151
≥ 21	27.8	1.82 (1.09-3.06)	0.023
Employment type			
Tenured/public service	27.1	1.00	
Temporary/contracted	17.1	0.56 (0.37-0.82)	0.003
Weekly workload (hours)			
≤ 39	23.9	1.00	
≥ 40	24.4	1.03 (0.73-1.44)	0.879
Excessive classroom noise			
No	17.4	1.00	
Yes	30.5	2.09 (1.48-2.94)	0.000
Classroom indiscipline			
No	17.2	1.00	
Yes	30.3	2.10 (1.48-2.96)	0.000
Physical and/or verbal violence			
No	15.5	1.00	
Yes	28.7	2.20 (1.50-3.23)	0.000
Effort-reward imbalance			
No	13.7	1.00	
Yes	27.7	2.41 (1.55-3.77)	0.000
Overcommitment to work			
No	15.7	1.00	
Yes	31.4	2.45 (1.72-3.49)	0.000
Job dissatisfaction			
No	19.9	1.00	
Yes	34.5	2.13 (1.50-3.01)	0.000
Self-rated health			
Positive	22.2	1.00	
Negative	25.6	1.21 (0.86-1.69)	0.268
Self-rated quality of life			
Positive	17.3	1.00	
Negative	33.6	2.43 (1.73-3.41)	0.000
Noncommunicable chronic disease			
No	21.4	1.00	
Yes	29.5	1.53 (1.09-2.16)	0.014
Diagnosis of depression			
No	18.0	1.00	
Yes	46.1	3.90 (2.70-5.63)	0.000
Diagnosis of anxiety			
No	13.1	1.00	
Yes	36.4	3.78 (2.64-5.42)	0.000
Psychological/psychiatric follow-up			
No	17.4	1.00	
Yes	41.2	3.32 (2.34-4.71)	0.000
Use of antidepressants/tranquilizers			
No	14.5	1.00	
Yes	43.2	4.48 (3.16-6.35)	0.000
Sickness absence			
No	14.0	1.00	
Yes	33.3	3.08 (2.14-4.42)	0.000
Poor sleep quality			
No	9.7	1.00	
Yes	28.7	3.73 (2.22-6.27)	0.000
Suspected voice disorder			
No	18.1	1.00	
Yes	38.9	2.88 (2.03-4.07)	0.000
Healthy eating pattern			
No	26.7	1.58 (1.09-2.30)	0.016
Yes	18.7	1.00	
Regular physical activity			
No	24.8	1.06 (0.76-1.49)	0.717
Yes	23.6	1.00	

OR = odds ratio.

The final adjusted model indicated a higher likelihood of occupational disease diagnosis among teachers aged 40-49 years, those who reported experiencing physical and/or verbal violence from students, those dissatisfied with their work, individuals using antidepressant and/or tranquilizing medications, those who had taken sick leave in the previous year, and those with suspected voice disorders (Table 3).

**Table 3:** Final adjusted multiple logistic regression model of factors associated with the diagnosis of occupational disease. Public basic-education teachers in Minas Gerais, 2024 (n = 767)

Variable	Adjusted OR (95%CI)	p-value
Age (years)		
≤ 39	1.00	
40-49	1.94 (1.21-3.10)	0.006
≥ 50	1.28 (0.79-2.09)	0.315
Physical and/or verbal violence		
No	1.00	
Yes	1.65 (1.08-2.51)	0.020
Job dissatisfaction		
No	1.00	
Yes	1.67 (1.14-2.46)	0.009
Use of antidepressants and/or tranquilizers		
No	1.00	
Yes	3.36 (2.31-4.88)	0.000
Sickness absence		
No	1.00	
Yes	1.87 (1.26-2.79)	0.002
Suspected voice disorder		
No	1.00	
Yes	1.80 (1.22-2.64)	0.003

Hosmer-Lemeshow test = 13.12. p-value = 0.217. Pseudo R^2^ = 14.9%.

OR = odds ratio.

## DISCUSSION

Approximately one quarter of the teachers investigated reported a diagnosis of an occupational disease. In the final model, odds were higher among teachers aged 40-49 years, those who reported episodes of physical and/or verbal violence perpetrated by students, those dissatisfied with their work, users of antidepressants and/or tranquilizers, those with sickness absence in the previous year, and those with suspected voice disorders.

The observed frequency differs from findings in a study of 314 faculty members at the Universidade Estadual de Feira de Santana, which reported a prevalence of 72.6%.^20^ This discrepancy may reflect contextual and methodological differences - including measurement instruments and criteria for defining “occupational complaint” - as well as sample profiles that differ in working conditions and institutional support. Despite these differences, both results underscore the need for continuous monitoring of teachers’ working conditions and effective strategies for health promotion and protection.^20^ Globally, an estimated 2.8 million deaths in 2017 (about 5% of all deaths) were attributable to work, 86% of which were related to occupational diseases.^2^

The higher odds in the 40-49-year group are consistent with an analytical review of cross-sectional studies of teachers from public and private systems across educational levels.^21^ In that literature, occupational conditions are more frequent around age 40, possibly reflecting longer exposure to overload factors.^21^

The association between school violence and occupational illness is also supported by prior evidence. Among public-school teachers in São Paulo, 50.5% reported physical or verbal aggression, which was linked to greater physical and mental exhaustion.^22^ Such experiences affect the illness process and the quality of classroom work.

Job dissatisfaction was associated with the outcome. In adverse contexts, pedagogical practice requires reconfigured planning and improvisation that alter content and expectations, favoring persistent dissatisfaction and feelings of frustration and helplessness, with potential effects on career retention.^5^,^7^

Use of antidepressants and/or tranquilizers was positively associated with the diagnosis of occupational disease. Studies of primary, secondary, and higher-education teachers in Belo Horizonte indicate that mental disorders are a leading cause of leave (15%).^5^ An investigation in municipal public schools in the metropolitan region of Porto Alegre likewise identified mental disorders among the most prevalent reasons for leave (11%).^11^

Sickness absence in the previous year was associated with the outcome, aligning with literature that identifies absenteeism as an indicator of work-related illness, particularly in high-demand occupations.^21^ High frequencies of absence may signal adverse working conditions - such as heavy workloads, performance pressure, classroom indiscipline, and lack of recognition - factors linked to worsening worker health and formal recognition of occupational diseases.^9^,^10^

Suspected voice disorders were also associated with the diagnosis. In a cross-sectional study of Brazilian basic-education teachers, voice disorder was the leading reason for leave (17.7%), followed by emotional problems (14.5%); high noise levels, poor acoustics, and overcrowded classrooms were among the main risk factors for vocal dysfunction.^9^

Formulating and implementing public policies to reduce socioeconomic vulnerabilities and improve institutional teaching conditions are essential for valuing teachers and reducing absenteeism due to occupational diseases.^10^ Balancing demands with professional recognition should guide investments in school infrastructure and organization, with implications for the health and quality of life of educators.^9^

This study has limitations. The cross-sectional design precludes causal inference. Self-reported measures may introduce recall bias, and internet-based data collection may lead to selection bias. In addition, the outcome question did not distinguish specific types of diagnosed occupational diseases. Web-based surveys do not ensure equiprobable sampling or precise estimates of response rates; however, we performed a sample-size calculation assuming an infinite population and outcome prevalence to guarantee a minimum n representative of Minas Gerais.

Strengths include the sociodemographic profile’s compatibility with basic-education teachers in Brazil, statewide coverage - including rural regions - adequate sample size, use of validated instruments, and rigorous methodological procedures, as well as institutional support from SEE-MG for dissemination and survey delivery.

## CONCLUSIONS

The occurrence of diagnosed occupational disease among basic-education teachers was multifactorial. Student-perpetrated physical and/or verbal violence, job dissatisfaction, use of psychotropic medications, health-related absences, and suspected voice disorders highlight the central role of working conditions and workplace relationships in teachers’ illness processes, which involve not only physical demands but also emotional and psychological components.

The association between school violence and higher occurrence of occupational diseases points to the need for strategies that promote safe school environments and support teachers’ mental health. Likewise, job dissatisfaction and use of psychotropic drugs underscore the urgency of policies for professional valuation, recognition, and well-being at work. The high frequency of voice disorders reinforces the importance of specific interventions, such as improving classroom acoustics and implementing vocal-hygiene programs.

Ongoing monitoring and research on teachers’ health are essential to guide effective prevention and health-promotion actions in educational settings. Investing in teachers’ health also means investing in education quality and social development.
